# 

**Published:** 1872-12-15

**Authors:** 


### Medical Examiner.

A Semi-Monthly Journal of Medical Sciences.

No. 24. CHICAGO, DECEMBER 15, 1872. Vol. XIII.

